# AI-assisted learning in exercise physiology: a quasi-experimental study using PhysioExercise GPT

**DOI:** 10.3389/fphys.2026.1894046

**Published:** 2026-09-08

**Authors:** Roque Ribeiro Da Silva Junior, Larissa Nayara de Souza, Gilson Aquino Cavalcante, Fernando Libralino Fernandes, José Antônio da Silva Júnior, Rackel Gurgel Hipólito, Fausto Guzen, Edson Fonseca Pinto, Maria Irany Knackfuss, Thales Allyrio Araújo De Medeiros Fernandes

**Affiliations:** University of the State of Rio Grande do Norte, Mossoro, Brazil

**Keywords:** artificial intelligence, educational technology, exercise physiology, health education and awareness, teaching

## Abstract

This study evaluated the applicability and effectiveness of PhysioExercise GPT, an artificial intelligence tool based on the ChatGPT architecture, developed to support the teaching of exercise physiology to undergraduate physical education students. A longitudinal quasi-experimental study was conducted involving a total of 64 students. Thirty-two students used the AI tool through a structured educational approach that incorporated personalized support, immediate feedback, and learning activities based on Bloom’s Taxonomy, while a control group of 32 students received traditional instruction. The results showed that both groups improved over time; however, the AI-assisted group achieved significantly greater learning gains (*p* = 0.002). The intervention group demonstrated a mean improvement of 3.38 points compared with 1.97 points in the control group, corresponding to a large effect size (Hedges’ *g* = 0.80). Analysis of covariance (ANCOVA) confirmed that the superiority of the AI-assisted group remained significant after adjusting for baseline performance. The findings indicate that the structured use of PhysioExercise GPT functions as an effective digital tutor, promoting active learning and enhancing the retention of complex concepts. Although the study has limitations, including the absence of randomization, the results suggest that AI represents a robust complementary educational tool capable of improving academic performance in health education.

## 1 Introduction

Health education has undergone a significant transformation driven by the integration of digital technologies aimed at enhancing teaching and learning processes. Exercise physiology constitutes a fundamental discipline in the education and training of health professionals, requiring not only the acquisition of theoretical knowledge but also the development of analytical reasoning and practical competencies. However, traditional teaching approaches in this field often rely on passive instructional methods, which may limit student engagement and hinder the effective understanding and retention of complex physiological concepts. Moreover, classroom environments are characterized by diverse student profiles and distinct learning styles, which may contribute to disparities in learning outcomes and difficulties among students who may not feel adequately supported by the teaching methodologies employed (Aguiar, 2023; Caitano et al., 2024; Cardoso et al., 2023).

Artificial intelligence (AI) has emerged as a promising educational technology. Although AI had already been used to some extent before the widespread adoption of generative AI, its use became substantially more widespread following the launch of ChatGPT in November 2022 (Fazlollahi et al., 2022; Regis et al., 2025). This development was accompanied by a growing diversity of applications, including the potential to enhance teaching and learning through personalized and adaptive learning environments. AI-based educational systems can function as intelligent virtual tutors by providing immediate feedback, adaptive questioning, and individualized learning pathways, thereby promoting knowledge retention and improving student performance (Favero, 2024; Rädel-Ablass et al., 2025). Evidence from medical education and the education of health professionals indicates that AI-assisted learning may improve academic performance and competency development; however, reported outcomes remain heterogeneous across different educational contexts and study designs (Reis et al., 2024; Yildiz et al., 2026).

Evidence regarding the effectiveness of AI-based educational tools specifically developed for teaching exercise physiology remains limited. Most published studies have focused on broader applications within medical education, with relatively few investigations examining the systematic integration of AI into physiology instruction to improve learning outcomes. Furthermore, there is a paucity of evidence regarding the applicability of these tools in authentic undergraduate educational settings, particularly when evaluated using longitudinal comparative study designs.

To address this gap, PhysioExercise GPT was developed as a personalized educational tool designed to support the teaching of exercise physiology through individualized interactions, guided questioning, and adaptive feedback grounded in Bloom’s Taxonomy. Therefore, the present study aimed to evaluate the applicability of PhysioExercise GPT and to compare AI-assisted instruction with conventional teaching in terms of academic performance among undergraduate Physical Education students.

## 2 Methodology

### 2.1 Study design

This was a longitudinal study with a quasi-experimental design, conducted to evaluate the applicability and effectiveness of an artificial intelligence-based educational intervention. The study was designed and reported in accordance with the Strengthening the Reporting of Observational Studies in Epidemiology (STROBE) guidelines.

Given the characteristics of the educational setting, participants were allocated into groups within the same academic cohort based on operational and academic feasibility. Although this approach may introduce selection bias, efforts were made to ensure comparability between groups by maintaining equivalent academic conditions, including the same curriculum, instructor, and learning environment.

In addition, statistical adjustment procedures were applied, including analysis of covariance (ANCOVA), using baseline values as covariates to reduce potential initial differences between groups and approximate an adjusted comparative model.

### 2.2 Setting, study period, and follow-up

The study was conducted at the Faculty of Physical Education (FAEF) of the State University of Rio Grande do Norte (UERN), Brazil, during regular Exercise Physiology classes. The follow-up period lasted approximately six months (one academic semester). Two main assessment time points were defined: baseline (T0) and post-intervention (T1). In addition, student engagement and participation were monitored throughout the semester.

### 2.3 Participants, sample, and group allocation

A *post hoc* power analysis was conducted using G*Power software (version 3.1.9.4). Considering an effect size of 0.80, α = 0.05, and a total sample of 64 participants (32 per group), the achieved statistical power was 0.83, indicating adequate power to detect between-group differences.

Participants were allocated into two groups (n = 32 each): the PhysioExercise GPT group, which used the AI-based tool as structured learning support, and the Conventional Teaching (control) group, which followed traditional teaching methods without structured use of AI tools.

The allocation procedure adopted was simple randomization. Participants were assigned to the groups through a simple lottery procedure. Each participating student received an identification code consisting of an Arabic numeral instead of their name. The randomization process was conducted by an external evaluator who had no contact with the study sample. Participants were sequentially allocated by drawing one identification number for the AI intervention group and the next for the control group until the total sample of 64 participants was reached, with 32 individuals assigned to each group. This procedure resulted in a quasi-experimental parallel-group design. Given the shared classroom environment, complete separation between groups was not feasible, which may have introduced a risk of contamination bias.

### 2.4 Eligibility criteria and follow-up

Students regularly enrolled in the 5th semester, actively participating in the course, and who signed the informed consent form were included. Students who did not complete the assessments (T0 and/or T1), withdrew from the course, or failed to meet minimum participation requirements were excluded.

### 2.5 Intervention and exposure characterization

The independent variable (exposure) was defined as the structured use of PhysioExercise GPT as a learning support tool. PhysioExercise GPT is an artificial intelligence system based on the ChatGPT architecture, specifically developed for teaching Exercise Physiology. The system was built using a curated scientific database (PubMed full-text documents) and pedagogical strategies grounded in Bloom’s taxonomy.

The tool enables adaptive quizzes, personalized feedback, simulation of practical scenarios, and structured cognitive progression. A seven-day familiarization period was implemented before the intervention to allow participants to adapt to the tool.

Students in the intervention group were instructed to use the tool for approximately 20–30 minutes per session, at least three times per week, throughout the semester. The instructor maintained the same curriculum, teaching schedule, and evaluation criteria for both groups, ensuring that the only difference between them was the structured use of the AI tool. In the intervention group, the instructor acted as a facilitator of technology use, while in the control group, traditional teaching strategies were applied, including lectures and guided activities. Control group participants were instructed not to use AI-based tools during the study period; however, complete control over this condition was not feasible, and this is acknowledged as a methodological limitation.

### 2.6 Variables and potential confounders

The primary outcome was performance in Exercise Physiology, measured by total scores (0–10 points) in standardized assessments. Potential confounding variables included baseline academic performance, attendance and participation, extracurricular study time, digital literacy, use of external AI tools, student motivation, and possible interaction between groups. These variables were not experimentally controlled and are recognized as inherent limitations of the study design.

### 2.7 Measurement instruments and data collection procedures

Two standardized assessments (T0 and T1) were administered, aligned with the course syllabus. Each assessment consisted of 10 multiple-choice questions, with a maximum score of 10 points.

Although the number of items was limited, the questions were developed using a problem-based learning (PBL) approach, as proposed by Borochovicius and Tortella (2014), and distributed across different cognitive levels of Bloom’s taxonomy (recall, comprehension, application, analysis, and evaluation), aiming to ensure content validity and conceptual coverage.

### 2.8 Strategies to reduce bias

To minimize measurement bias, assessments were anonymized and graded by blinded evaluators. Each test was independently scored by two evaluators, with final scores calculated as the average. Discrepancies were resolved by a third evaluator. To reduce performance bias, both groups were exposed to the same curriculum and educational environment, differing only in the structured use of PhysioExercise GPT.

### 2.9 Statistical analysis

Descriptive analyses were initially conducted (mean, standard deviation, median, and dispersion measures). Data normality was assessed using the Shapiro–Wilk test, and homogeneity of variances using Levene’s test.Within-group comparisons (pre vs. post) were performed using paired t-tests. Between-group comparisons of gains (Δ = post − pre) were conducted using independent samples t-tests. Additionally, analysis of covariance (ANCOVA) was performed, using post-intervention scores as the dependent variable and baseline values as covariates, in order to adjust for initial differences between groups and increase analytical robustness. Confidence intervals (95%) and effect sizes (Hedges’ g) were reported. Statistical analyses were conducted using SPSS software (version 20), with a significance level set at p < 0.05.

### 2.10 Ethical considerations

The study was conducted in accordance with the ethical principles of the Declaration of Helsinki and Brazilian National Health Council Resolutions No. 466/12 and No. 510/16. The study was approved by the Research Ethics Committee of UERN (CAAE: 80370924.2.0000.5294; approval number 6.959.230). All participants provided informed consent.

## 3 Results

### 3.1 PhysioExercise GPT

PhysioExercise GPT (version GPT-5.6) is a specialized version of ChatGPT specifically developed to support the teaching and learning of Exercise Physiology. The development of the tool involved three main components: (1) selection and organization of the knowledge base; (2) development of prompts and pedagogical interaction strategies; and (3) development and evaluation of the instrument used to assess student performance.

It is important to clarify that, during the intervention period conducted in 2024, PhysioExercise GPT used GPT-4.5 as its base model, and this was the version made available to participants throughout the entire data collection period. Access to the tool occurred between 22 July and 27 October 2024. Following the completion of the intervention and data collection, PhysioExercise GPT underwent subsequent updates, including a version based on GPT-5.0 and, subsequently, the current version based on GPT-5.6. It should be emphasised that these modifications were implemented only after the end of the experimental period and, therefore, did not affect participants’ exposure to the tool or the results obtained in the present study. Accordingly, all analyses presented in this study refer exclusively to the GPT-4.5-based version of PhysioExercise GPT used during the intervention period.

The knowledge base was constructed using documents retrieved from the National Library of Medicine (PubMed) with the search term “Physiology exercise” and the filters “Books and Documents” and “Free full text.” Following the search and selection process, 58 documents published between 1990 and 2025 were incorporated into the tool’s knowledge base. In addition, educational frameworks grounded in Problem-Based Learning (PBL) were included, encompassing publications from 2004 to 2025.

The materials used to construct PhysioExercise GPT were selected by three experts holding doctoral degrees in Physiological Sciences. The retrieved documents were independently and blindly assessed using the Rayyan QCRI platform. Each reviewer classified the materials as either eligible or ineligible for inclusion in the knowledge base. Documents receiving favorable agreement from at least two experts were included. In cases of disagreement among reviewers, a senior physiology expert conducted an additional assessment to resolve the discrepancy while maintaining the blinded evaluation process.

**Figure 1:**
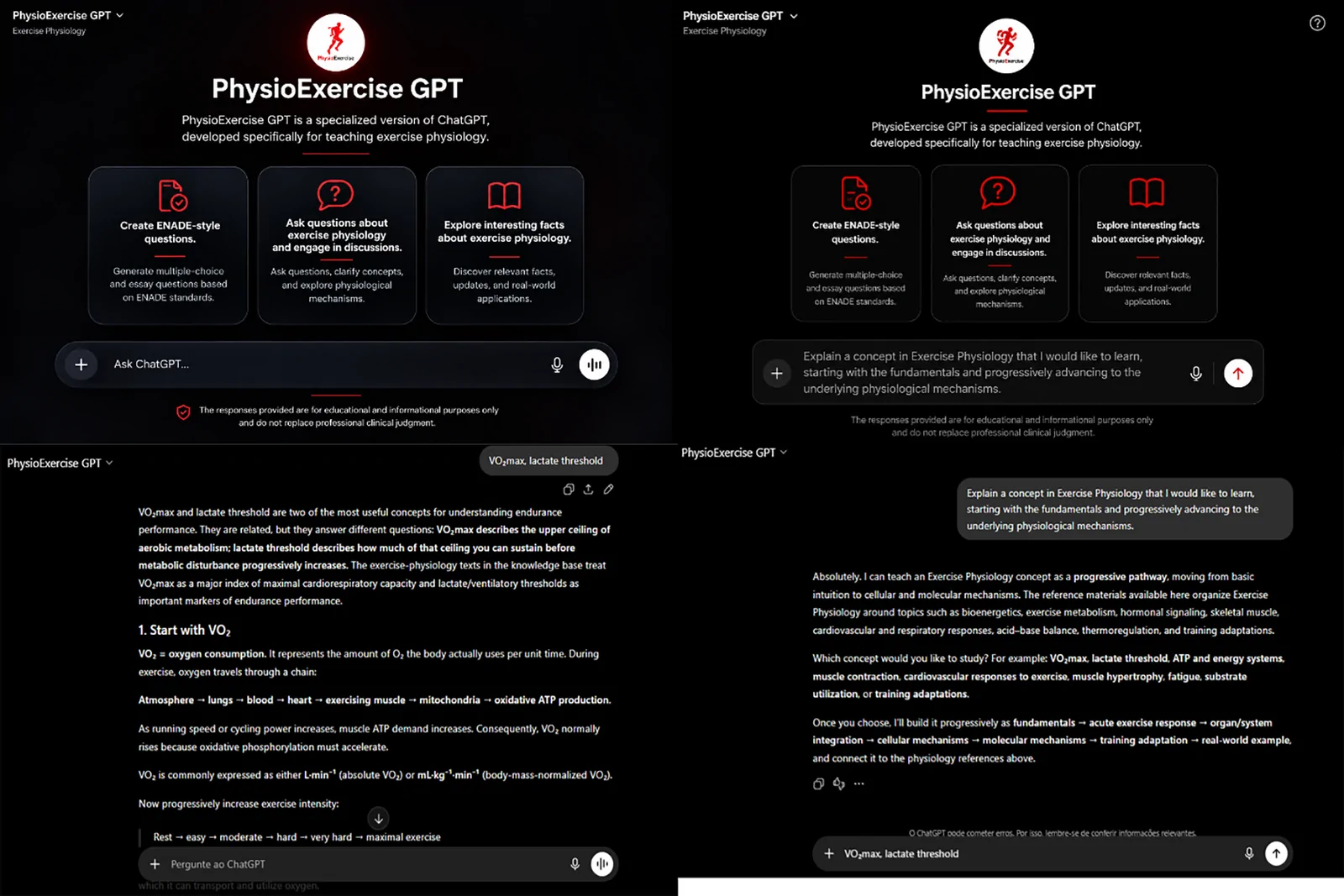
PhysioExercise GPT interface. Home screen of PhysioExercise GPT showing the “Ask something” text box and the corresponding icebreakers.

During the development of the tutor, ten core prompts and initial interaction scripts (icebreakers) were implemented to guide students in using the tool and to promote greater standardization of interactions **Table 1**. The prompts were structured to support educational activities based on Bloom’s Taxonomy and to address different levels of cognitive complexity, including remembering, understanding, applying, analyzing, and evaluating. The tool was also configured to provide educational feedback and contextualized problem-based scenarios related to Exercise Physiology. As shown in **Figure 1**.

### 3.2 Development and evaluation of the performance assessment instrument

The instrument used to assess student performance consisted of 10 multiple-choice questions. The questions were developed by the same physiology experts responsible for selecting the PhysioExercise GPT knowledge base. During instrument development, the items were evaluated in terms of scientific content accuracy, clarity of wording, appropriateness of the response alternatives, alignment with the educational objectives, and cognitive level according to Bloom’s Taxonomy. The prompts and the Bloom’s Taxonomy classification implemented in the study are presented in **Table 1**.

**Table 1:** PhysioExercise GPT prompts and Bloom’s taxonomy classification.

Prompt/icebreaker provided to students	Educational objective	Bloom’s taxonomy
“Explain a concept in Exercise Physiology that I would like to learn, starting with the fundamentals and progressively advancing to the underlying physiological mechanisms.”	Facilitate the identification, definition, and explanation of fundamental concepts.	Remember/Understand
“Explain how the body responds to the transition from rest to exercise and relate these responses to the maintenance of homeostasis.”	Relate exercise-induced physiological changes to the regulation of the internal environment.	Understand/Apply
“Teach me about bioenergetics as applied to exercise and explain how the energy systems contribute to ATP resynthesis during exercise of different intensities and durations.”	Apply concepts of energy metabolism to different exercise conditions.	Understand/Apply
“Present a problem-based scenario involving physical exercise and ask me to explain the expected cardiovascular and respiratory responses. Then provide feedback on my answer.”	Integrate cardiovascular and respiratory responses with the metabolic demands of exercise.	Apply/Analyze
“Create a case involving metabolism during exercise and ask me to analyze carbohydrate and lipid utilization according to exercise intensity and duration.”	Develop reasoning regarding the selection and utilization of energy substrates.	Apply/Analyze
“Present an exercise scenario and ask me to analyze hormonal responses and their relationship with energy substrate availability.”	Relate neuroendocrine responses, exercise, and metabolic regulation.	Apply/Analyze
“Create a problem involving fatigue and muscular performance and ask me to relate skeletal muscle structure and function, energy production, and motor control.”	Integrate neuromuscular and metabolic knowledge to explain performance.	Analyze
“Compare two exercise conditions with different intensities or durations and ask me to identify and justify differences in the physiological responses.”	Encourage comparison, integration of physiological systems, and identification of causal relationships.	Analyze/Evaluate
“Present an applied Exercise Physiology case and ask me to propose a physiological explanation for the reported findings, justifying my response based on the underlying mechanisms.”	Promote evidence-based decision-making and integration of physiological knowledge.	Analyze/Evaluate
“Assess my knowledge of Exercise Physiology using a problem-based scenario. First, allow me to answer without revealing the solution; then analyze my response, identify correct elements and knowledge gaps, and provide explanatory feedback.”	Promote formative assessment, self-regulated learning, and individualized feedback.	Evaluate

Source: Prepared by the author (2026).

To characterize the cognitive scope of the instrument, the ten questions were prospectively classified according to Bloom’s Taxonomy based on the predominant cognitive process required for their resolution. The instrument comprised two questions at the understanding level (20%), three at the applying level (30%), three at the analyzing level (30%), and two at the evaluating level (20%). Thus, eight of the ten items (80%) required cognitive processes extending beyond conceptual understanding, including the application of physiological knowledge to contextualized situations, data interpretation, integration of physiological responses, and evaluation of exercise-related explanations. The items covered bioenergetics, exercise metabolism, substrate utilization, hormonal regulation, cardiovascular and respiratory responses, and physiological adaptations to training.

### 3.3 Content validity of the instrument

The questions and their respective response alternatives were independently evaluated by three experts holding doctoral degrees in Physiological Sciences, who were also involved in selecting the scientific knowledge base and developing the educational components of PhysioExercise GPT. The experts assessed each item with respect to clarity, relevance, representativeness of Exercise Physiology content, and appropriateness of the cognitive level assigned according to Bloom’s Taxonomy.

The evaluation showed unanimous agreement among the three experts (100%) regarding the adequacy of the items included in the T0 and T1 instruments. Based on the proportion of experts who rated each item as adequate, an item-level Content Validity Index (I-CVI) of 1.00 was obtained, indicating complete agreement among the evaluators.

Two versions of the assessment instrument were used, designated T0 (pre-intervention) and T1 (post-intervention), each consisting of 10 multiple-choice questions with five response options (A–E) and a single correct answer. The instruments were designed as parallel forms with planned equivalence in both content and cognitive demand. Accordingly, each T0 item had a corresponding T1 item assessing the same physiological domain, learning objective, and cognitive level according to Bloom’s Taxonomy.

In both versions, two questions (20%) were classified at the understanding level, three (30%) at the applying level, three (30%) at the analyzing level, and two (20%) at the evaluating level. The items addressed bioenergetics, energy metabolism, substrate utilization, hormonal regulation, cardiovascular and respiratory responses, homeostasis, and physiological adaptations to training. These domains correspond to the main content areas represented in the literature used to construct the PhysioExercise GPT knowledge base. According to **Table 2**.

**Table 2:** Characterization and distribution of the assessment items.

Questions	Bloom’s Taxonomy	Number of questions	%
1–2	Understand	2	20%
3–5	Apply	3	30%
6–8	Analyze	3	30%
9–10	Evaluate	2	20%
Total	—	10	100%

Source: Prepared by the author (2026). The complete assessment instruments are available in the **Supplementary Materials** accompanying this article.

To ensure transparency and reproducibility, the complete T0 and T1 instruments are provided in the **Supplementary Materials**. For each item, the materials include the question stem, five response alternatives, correct answer, physiological content assessed, corresponding cognitive level according to Bloom’s Taxonomy, and planned difficulty classification. Items were scored dichotomously (0 = incorrect response; 1 = correct response), yielding a total score ranging from 0 to 10 points at each assessment time point.

The study sample comprised 64 participants, of whom 71% were female and 29% were male. The mean age was 21.3 ± 3.21 years, and the participants reported a mean daily study time of 15.2 ± 4.6 minutes. This assessment was structured around two main domains. The first corresponds to the Computer and Information Literacy (CIL) Practical Skills Test, comprising interactive activities that simulate real-world situations involving the collection, management, creation, and exchange of information in digital environments. The second domain refers to Computational Thinking (CT), which is intended to assess participants’ ability to develop logical and algorithmic solutions to different problems through the use of digital tools. Participants’ performance in each proposed task is converted into a numerical score, with 750 points representing the maximum score established for the assessment. Proficiency is classified into four levels: Level 1, up to 492 points; Level 2, from 492 to 576 points; Level 3, from 576 to 680 points; and Level 4, above 680 points. Accordingly, the scores obtained enable participants to be classified according to different levels of proficiency in the digital competencies assessed.

The proposed tasks range from basic operational skills to more complex competencies related to the functional and strategic use of digital technologies. As an example of an operational skill, participants may be required to click on the “Insert Image” option, navigate through folders on a simulated computer, and select a specific photograph, such as an image of a recycling bin, to insert into a digital post. This type of activity enables the assessment, within a practical and interactive context, of participants’ ability to locate, select, manipulate, and use digital information and resources. In this context, the group achieved a mean digital literacy score of 581.4 ± 12.1 points, corresponding to Level 3 on the International Computer and Information Literacy Study (ICILS) scale.

### 3.4 Access records and adherence rate

After the implementation of PhysioExercise GPT, usage records were monitored through the OpenAI dashboard. These records made it possible to characterize the use of the tool by participants in the intervention group over a 26-week follow-up period. Considering the minimum recommendation of two uses per week, a planned dose of 52 uses per participant was established. A minimum adherence threshold corresponding to 75% of the planned dose was then defined. Accordingly, participants who completed 39 or more uses during the intervention period were considered adherent to the protocol. The overall usage records for PhysioExercise GPT are presented in **Table 3**.

**Table 3:** Access records and adherence rate.

System record	Results
Number of participants	32
Total number of interactions	1.402
Mean number of interactions per participant	43,81
Mean usage time*	23,5± 6,3
Mean number of days with recorded interactions	93 ± 11,1
Mean number of messages sent per participant to PhysioExercise GPT*	114 ± 12,3
Planned dose per participant	52 uses
Minimum number of uses required for adherence ≥ 75%	39 uses
Mean usage rate relative to the planned dose	84,25%

**Source:** Author’s own elaboration (2026).

### 3.4 Applicability of PhysioExercise GPT

Data were collected from students enrolled in the fifth semester of the Physical Education program at UERN. Following classroom recruitment and the provision of informed consent, participants underwent a seven-day familiarization period with the tool. Subsequently, pre-intervention (T0) and post-intervention (T1) assessments were administered, each consisting of ten multiple-choice questions.

Importantly, access to PhysioExercise GPT was restricted to students assigned to the AI intervention group. Although the use of other AI tools outside the study environment could not be fully controlled, participants assigned to the control group did not have access to the PhysioExercise GPT tool used in the intervention. Residual analysis using the Shapiro–Wilk test showed no significant deviation from normality (W = 0.985, p = 0.642). Similarly, Levene’s test did not indicate heterogeneity of variances between groups, F(1, 62) = 0.694, p = 0.408, supporting the assumption of homogeneity of variance.

For the ANCOVA, the assumptions underlying the model were assessed prior to analysis. An approximately linear relationship was observed between baseline performance (T0) and post-intervention performance (T1). Residual analysis showed no significant deviation from normality according to the Shapiro–Wilk test (W = 0.985, p = 0.642), and Levene’s test supported homogeneity of variances, F(1, 62) = 0.694, p = 0.408. In addition, the homogeneity of regression slopes was assessed by testing the interaction between group and baseline performance (Group × T0). The interaction was not statistically significant, F(1, 60) = 0.116, p = 0.735, indicating that the relationship between T0 and T1 did not differ significantly between groups and supporting the assumption of homogeneous regression slopes.

Both groups showed statistically significant improvements from T0 to T1. In the PhysioExercise GPT group, the mean score increased from 3.88 ± 1.41 at baseline to 7.25 ± 1.19 after the intervention, corresponding to a mean gain of 3.38 ± 1.60. This within-group improvement was statistically significant, t(31) = 11.92, p < 0.001. Similarly, the control group showed an increase from 3.09 ± 1.67 at T0 to 5.06 ± 1.50 at T1, with a mean gain of 1.97 ± 1.86, which was also statistically significant, t(31) = 6.00, p < 0.001 (**Table 4**).

**Table 4:** Descriptive statistics and within-group comparisons of pre- and post-intervention performance.

Group	n	T0 (mean ± SD)	T1 (mean ± SD)	Δ (mean ± SD)	*t* (df)	*p*-value
PhysioExercise GPT	32	3.88 ± 1.41	7.25 ± 1.19	3.38 ± 1.60	11.92 (31)	< 0.001
Control	32	3.09 ± 1.67	5.06 ± 1.50	1.97 ± 1.86	6.00 (31)	< 0.001

Source: Prepared by the author (2026). Data are presented as mean ± standard deviation (SD). T0 represents pre-intervention performance, T1 represents post-intervention performance, and Δ represents the individual change calculated as T1 − T0. Within-group comparisons between T0 and T1 were performed using paired-samples *t*-tests.

The PhysioExercise GPT group exhibited a greater mean performance gain (3.38 ± 1.60) than the control group (1.97 ± 1.86), resulting in a mean between-group difference of 1.41 points (95% CI: 0.54 to 2.27). The difference in gains was statistically significant, *t*(62) = 3.244, *p* = 0.002. The effect size was large (Hedges’ *g* = 0.80), indicating a substantial magnitude of the between-group difference in performance gains (**Table 5**).

**Table 5:** Intergroup comparison of performance gains (Δ).

Δ = T1 − T0	GPT (mean ± SD)	Control (mean ± SD)	Difference (GPT − control)	95% CI	*t* (df)	*p*-value	Hedges’ *g*
Gain (Δ)	3.38 ± 1.60	1.97 ± 1.86	1.41	0.54 to 2.27	3.244 (62)	0.002**	0.80

Comparison of performance gains (Δ = post-intervention − pre-intervention) between the PhysioExercise GPT group (*n* = 32) and the control group (*n* = 32). Data are presented as mean ± standard deviation (SD), together with the mean between-group difference (GPT − Control), 95% confidence interval (95% CI), independent-samples *t*-test statistic, degrees of freedom (df), and Hedges’ *g* effect size. ** indicates statistical significance at *p* < 0.01.

An analysis of covariance (ANCOVA) revealed a statistically significant effect of group on post-intervention performance after adjustment for baseline performance (T0), *F*(1, 61) = 34.73, *p* < 0.001, partial η^2^ = 0.363. This finding indicates that significant between-group differences in post-intervention performance remained after controlling for baseline scores.

Baseline performance (T0), included as a covariate, was also significantly associated with post-intervention performance, *F*(1, 61) = 5.65, *p* = 0.021, partial η^2^ = 0.085. The effect size associated with group was greater than that associated with the covariate, indicating that group membership accounted for a larger proportion of the variance in post-intervention performance (**Table 6**).

**Table 6:** Analysis of covariance (ANCOVA) for post-intervention performance.

Source of variation	SS	df	MS	*F*	*p*-value	Partial η^2^
Group	59.34	1	59.34	34.73	< 0.001*	0.363
T0 (covariate)	9.65	1	9.65	5.65	0.021*	0.085
Error	104.23	61	1.71	—	—	—

ANCOVA was performed to compare post-intervention performance between groups while adjusting for baseline performance (T0). SS = sum of squares; MS = mean square; df = degrees of freedom; partial η^2^ = partial eta squared. * indicates statistical significance at *p* < 0.05.

## 4 Discussion

The present study demonstrated that the structured use of PhysioExercise GPT was associated with a significant improvement in student performance, with higher mean gains observed in the intervention group (Δ = 3.38 ± 1.60) compared to the control group (Δ = 1.97 ± 1.86), along with a statistically significant between-group difference (p = 0.002) and a large effect size (Hedges’ g = 0.80). These findings suggest that the use of the tool may be related to meaningful gains in the learning process in Exercise Physiology. However, due to the quasi-experimental design, these results should be interpreted as associations, and causal inference cannot be established.

The magnitude of the effect observed in the present study is consistent with recent evidence in the literature. Peng et al. (2025), synthesizing 12 studies (n = 824), reported the superiority of AI-assisted education compared to traditional teaching (SMD = 2.06), while Mo et al. (2026) identified a high overall effect (Hedges’ g = 1.14), albeit with considerable variability. In this context, the effect size observed in the present study (g = 0.80) aligns with these findings, suggesting that AI-based interventions may produce relevant educational impacts, although these effects remain context-dependent.

The greater gains observed in the PhysioExercise GPT group may be related to specific features of the tool, such as immediate feedback, progressive adaptation of difficulty levels, and content organization based on Bloom’s taxonomy. These elements promote active and self-regulated learning, which are widely recognized as key determinants of academic performance. In this regard, Sriram et al. (2025) highlight that AI-based systems function as digital tutors, providing personalized feedback and enhancing engagement, which may partially explain the results observed in this study.

Furthermore, the use of problem-based scenarios and applied situations may have contributed to the higher gains observed in the intervention group. Ramsamooj et al. (2025) reported that AI-based tools improved interpretative abilities among health students, although without statistically significant differences across all outcomes. Similarly, in the present study, although both groups improved over time, the AI-assisted group demonstrated greater gains, suggesting a potential additional effect of the tool on the learning process.

On the other hand, the literature indicates that the effects of AI may vary depending on the type of outcome assessed. Ba et al. (2024), in a controlled study, found no significant differences in theoretical assessments but reported improvements in practical skills in the AI-assisted group. This finding is relevant for interpreting the present results, as the outcome assessed was predominantly theoretical, which may underestimate the tool’s impact on more applied domains.

Additionally, Li et al. (2025), in a meta-analysis including 786 participants, reported improved performance and enhanced learning experiences in AI-assisted groups. The present findings are consistent with this evidence, as higher performance gains were observed in the intervention group, even after statistical adjustment using ANCOVA, suggesting that the observed association is not solely explained by baseline differences.

Within the same context, but addressing competencies that complement AI supported learning, Yildiz and Nisanci (2026) conducted a randomised educational study involving 90 physiotherapy students. Participants were equally allocated to three groups: training supported by artificial intelligence, internet supported training combined with traditional instruction, and exclusively traditional instruction. Over eight weeks, the students analysed musculoskeletal clinical scenarios and developed assessment and treatment plans. Digital competence increased significantly in the groups that used artificial intelligence or internet resources, whereas no significant improvement was observed in the traditional group. Although clinical self-efficacy and attitudes towards artificial intelligence improved in all three groups, comparisons of change scores revealed a significant between-group difference only in digital competence (p < 0.001). No superiority was observed between the groups for clinical self-efficacy (p = 0.060) or attitudes towards artificial intelligence (p = 0.998). Therefore, these findings support the use of artificial intelligence as a complementary resource for developing digital competencies, although they do not yet demonstrate a consistent additional benefit regarding clinical competence.

Extending this critical perspective beyond the isolated use of artificial intelligence, Yıldız and Nişancı (2026) investigated the effects of different psychosocial rehabilitation teaching strategies on the competencies and attitudes of 99 physiotherapy students towards older adults and individuals with disabilities. Participants were randomised into three groups: psychosocial rehabilitation training combined with anatomy based practical activities, exclusively theoretical psychosocial rehabilitation training, and the conventional curriculum. Both intervention groups demonstrated significant improvements in psychosocial care competence and attitudes towards older adults and individuals with disabilities, whereas the control group showed no significant changes. Between-group comparisons demonstrated large effect sizes for psychosocial competence (η^2^ = 0.30), attitudes towards disability (η^2^ = 0.49), and attitudes towards ageing (η^2^ = 0.40). However, no significant difference was identified between theoretical training and training supplemented with a practical component. These results highlight the importance of systematically integrating psychosocial rehabilitation education into health sciences curricula, although they do not confirm the statistical superiority of the practical component.

Importantly, taken together, these findings indicate that structured educational interventions can promote the development of professional competencies, positive attitudes, and digital skills among rehabilitation students. Nevertheless, they also demonstrate that simply incorporating a practical component or an artificial intelligence tool does not necessarily ensure superiority over other structured educational strategies. In the study involving clinical scenarios supported by artificial intelligence, the main comparative benefit was observed in digital competence, whereas clinical self-efficacy followed a similar trajectory across the groups. These findings suggest that the educational effects of artificial intelligence depend on its integration with clearly defined pedagogical objectives, contextualised clinical cases, instructor guidance, and clinical reasoning activities. Therefore, the observed educational benefits should not be attributed exclusively to the technology itself.

Despite these promising findings, it is important to note that the observed gains cannot be attributed exclusively to the use of PhysioExercise GPT. Factors such as student motivation, engagement, extracurricular study time, and potential use of external AI tools may have influenced the results. Lam et al. (2022) emphasize that, although most studies report benefits of AI, many present methodological limitations, including small sample sizes and heterogeneous designs, which limit the generalizability of findings. In this regard, the present study shares similar limitations, reinforcing the need for cautious interpretation.

Methodological limitations include the quasi-experimental design without randomization, which may introduce selection bias; the absence of an *a priori* sample size calculation, which limits the assessment of statistical power; and the use of an instrument with only ten items, which may reduce measurement sensitivity. Additionally, the lack of strict control over confounding variables, such as digital literacy, motivation, and the use of external tools, may have influenced the results.

Despite these limitations, the observed effect size (g = 0.80) suggests practical relevance in the educational context, indicating that the tool may represent an effective complementary strategy to traditional teaching. Sallam (2023) highlights that AI-based systems have the potential to promote personalized learning and the development of critical thinking, while Sridharan and Sequeira (2024) emphasize their ability to adapt instruction to individual student needs. The present findings support these perspectives, demonstrating greater gains among students who used the tool in a structured manner.

Finally, the heterogeneity reported in the literature, as highlighted by Mo et al. (2026) suggests that the impact of AI is strongly influenced by the educational context, pedagogical design, and implementation strategies. Thus, the findings of this study should be interpreted as preliminary evidence that the structured use of PhysioExercise GPT may be associated with improvements in academic performance, highlighting the need for future randomized studies with larger samples and long-term outcomes to confirm these results.

## 5 Conclusion

The present study suggests that the structured use of a GPT-based tool for teaching Exercise Physiology may improve learning outcomes compared to conventional instruction. Although both groups demonstrated significant improvement over time, the AI-assisted group exhibited greater gains, and this effect remained significant even after adjustment for baseline performance, reinforcing the consistency of the findings. These results may be explained by the adaptive and interactive nature of the tool, which enables personalized feedback, sustained engagement, and the development of higher-order cognitive skills.

## Data Availability

The original contributions presented in the study are included in the article/**Supplementary Material**. Further inquiries can be directed to the corresponding author.
