# Supplementary material for: AI-assisted learning in exercise physiology: a quasi-experimental study using PhysioExercise GPT
Source: Front Physiol. 2026 Sep 8;17:1894046. doi: 10.3389/fphys.2026.1894046 (PMC13595339; doi:10.3389/fphys.2026.1894046)
Supplement: Supplementary file 1 [file SupplementaryFile1.docx]

T0 — PRE-INTERVENTION ASSESSMENT

This version includes item difficulty classifications as Easy, Moderate, Difficult, and Very Difficult. These classifications were not identified in the version administered to the students.

Question 1 — Understanding | Easy

During a lesson on bioenergetics, a student stated that the small amount of ATP stored in skeletal muscle would be sufficient to sustain prolonged physical activity. Considering the mechanisms of energy supply, select the correct alternative.

A) Stored muscle ATP alone is sufficient to sustain prolonged exercise.

B) ATP resynthesis depends exclusively on aerobic metabolism.

C) Sustained muscle contraction requires continuous ATP resynthesis through different metabolic pathways.

D) Phosphocreatine is the primary energy source during prolonged exercise.

E) ATP production occurs only when exercise demands exceed resting metabolism.

Question 2 — Understanding | Easy

When an individual transitions from rest to moderate-intensity running, the demand for oxygen delivery to the active muscles increases. Which of the following is an expected cardiovascular response?

A) A decrease in cardiac output.

B) An increase in heart rate and cardiac output.

C) Cessation of venous return.

D) An obligatory reduction in stroke volume from the onset of exercise.

E) A decrease in blood flow to the active musculature.

Question 3 — Applying | Moderate

One sprinter performs an all-out sprint lasting approximately 6 seconds, whereas another athlete runs continuously for 40 minutes at moderate intensity. Which interpretation is most appropriate?

A) Oxidative metabolism predominates equally during both activities.

B) The first activity has a greater relative contribution from the phosphagen system, whereas the second relies predominantly on oxidative metabolism.

C) Both activities rely exclusively on anaerobic glycolysis.

D) The first athlete uses only lipids, whereas the second uses only carbohydrates.

E) Energy system contributions are not influenced by exercise duration.

The literature included in the knowledge base specifically describes anaerobic and aerobic ATP production and the influence of exercise intensity and duration on metabolism.

Question 4 — Applying | Moderate

During an incremental cycle ergometer test, the workload progressively increases. To meet the increasing metabolic demand, which response is expected?

A) A simultaneous decrease in ventilation and cardiac output.

B) Maintenance of cardiorespiratory responses at resting levels.

C) An integrated increase in ventilation and cardiac output.

D) A reduction in heart rate proportional to the increase in workload.

E) Cessation of blood flow redistribution.

Question 5 — Applying | Moderate

During prolonged exercise, maintenance of blood glucose becomes important for sustaining physical effort. Which response is physiologically consistent with this situation?

A) An obligatory increase in insulin and inhibition of hepatic glucose mobilization.

B) No hormonal involvement in metabolism.

C) A reduction in the mobilization of energy substrates.

D) Hormonal adjustments that promote substrate availability and contribute to the maintenance of blood glucose.

E) Exclusive utilization of muscle proteins.

The knowledge base describes a reduction in insulin and increases in hormones such as epinephrine, norepinephrine, growth hormone (GH), glucagon, and cortisol during exercise, which are associated with maintaining the availability of glucose and other energy substrates.

Question 6 — Analyzing | Difficult

Two individuals have similar total energy expenditures. Individual A performed shorter, higher-intensity exercise, whereas individual B performed prolonged, moderate-intensity exercise.

Consider the following statements:

I. Exercise intensity can modify the relative contributions of carbohydrates and lipids.

II. Exercise duration can influence substrate selection.

III. Similar total energy expenditure necessarily indicates identical substrate utilization.

Select the correct alternative.

A) I only.

B) II only.

C) I and II only.

D) II and III only.

E) I, II, and III.

Question 7 — Analyzing | Difficult

Consider the following data:

Condition	HR	Stroke Volume

Rest	70 bpm	70 mL

Moderate exercise	130 bpm	100 mL

High-intensity exercise	175 bpm	105 mL

Considering the relationship among heart rate, stroke volume, and cardiac output, which conclusion is correct?

A) Cardiac output decreases as exercise intensity increases.

B) From moderate- to high-intensity exercise, the additional increase in cardiac output is primarily explained by the increase in heart rate.

C) Cardiac output remains constant.

D) Heart rate does not influence cardiac output.

E) Cardiac output is lower during moderate exercise than at rest.

Question 8 — Analyzing | Difficult

During an incremental exercise test, ventilation progressively increases and shows a proportionally greater rise at higher exercise intensities. Which interpretation is most appropriate?

A) Metabolic demand is decreasing.

B) Ventilation is unrelated to metabolism.

C) Ventilatory adjustments accompany metabolic demand and, at higher intensities, are also related to changes in acid–base balance.

D) An increase in CO₂ necessarily decreases ventilation.

E) The respiratory system ceases to contribute to homeostasis.

Question 9 — Evaluating | Very Difficult

Following aerobic training, a student showed a substantial improvement in performance. A classmate concluded that this improvement occurred exclusively because the lungs had developed a greater capacity to store oxygen.

Which is the most appropriate evaluation of this conclusion?

A) Correct, because aerobic performance depends exclusively on the lungs.

B) Correct, because cardiovascular adaptations do not contribute to training responses.

C) Incorrect, because adaptations to training are integrated and cannot be explained exclusively by pulmonary O₂ storage.

D) Correct only for young individuals.

E) Incorrect, because training does not produce physiological adaptations.

Question 10 — Evaluating | Very Difficult

During incremental exercise, heart rate, cardiac output, ventilation, and metabolic demand all increased. Three students interpreted these findings as follows:

I. Physiological systems respond entirely independently of one another.

II. These changes represent integrated responses that contribute to meeting the demands of exercise and maintaining homeostasis.

III. Maintaining homeostasis means keeping all physiological variables exactly at their resting values.

Select the most appropriate alternative.

A) I only.

B) II only.

C) III only.

D) I and III only.

E) I, II, and III.

T1 — POST-INTERVENTION ASSESSMENT

Question 1 — Understanding | Bioenergetics | Easy

Sustained contractile activity in skeletal muscle depends on the continuous availability of ATP. However, during exercise, different metabolic pathways may simultaneously contribute to ATP resynthesis.

The coexistence of these pathways means that:

A) Activation of oxidative metabolism immediately terminates anaerobic ATP production.

B) Each energy system functions independently and only becomes active after the preceding system has been depleted.

C) Energy-transfer systems interact continuously, with their relative contributions changing according to the metabolic demands of exercise.

D) The contribution of the energy systems is determined exclusively by the amount of ATP stored before exercise.

E) Exercise intensity and duration do not influence the relative contribution of different metabolic pathways.

Question 2 — Understanding | Homeostasis | Easy

During the transition from rest to exercise, heart rate, pulmonary ventilation, energy metabolism, and blood flow distribution undergo substantial changes. Despite these alterations, regulatory mechanisms continue to operate to maintain the internal environment.

From a physiological perspective, this phenomenon demonstrates that homeostasis:

A) Corresponds to the absolute constancy of all physiological variables.

B) Represents a dynamic equilibrium in which regulatory responses are continuously adjusted to the disturbances imposed by exercise.

C) Exists only at rest and is necessarily interrupted during any form of exercise.

D) Prevents cardiovascular and metabolic changes during exercise.

E) Depends exclusively on the nervous system and is independent of endocrine and metabolic responses.

Question 3 — Applying | Energy Metabolism | Moderate

During the transition from rest to constant-intensity exercise, skeletal muscle ATP demand increases immediately. However, oxidative metabolism requires physiological adjustments before its rate of energy production can increase sufficiently.

Considering the integration of the energy systems, which interpretation is most appropriate?

A) ATP production remains reduced until oxidative metabolism reaches the required level of energy production.

B) Anaerobic pathways contribute to meeting energy demand while aerobic ATP production adjusts to the increased requirement.

C) The oxidative system is activated only after phosphocreatine has been completely depleted.

D) Glycolysis ceases when oxygen is available in skeletal muscle.

E) Increased energy demand results in the exclusive use of phosphocreatine throughout the activity.

Question 4 — Applying | Cardiovascular Control | Moderate

During dynamic exercise, increased metabolic activity in the working muscles requires greater oxygen delivery. To meet this requirement, cardiac output changes and blood flow is redistributed.

Which physiological principle best explains this response?

A) Blood flow to all tissues increases by the same proportion.

B) The cardiovascular system adjusts cardiac output and blood flow distribution according to the different metabolic demands of the tissues.

C) An increase in heart rate necessarily reduces oxygen delivery to skeletal muscle.

D) Vascular resistance necessarily increases in all tissues during exercise.

E) Blood flow distribution is independent of local metabolic activity.

Question 5 — Applying | Hormonal Regulation of Metabolism | Moderate

During prolonged exercise, skeletal muscle continues to require energy substrates, while the body must simultaneously contribute to maintaining blood glucose levels.

In this context, hormonal regulation is important because it:

A) Prevents the mobilization of energy stores during exercise.

B) Promotes adjustments in the availability and utilization of energy substrates according to metabolic demand.

C) Causes skeletal muscle to rely exclusively on blood glucose.

D) Eliminates the need for hepatic regulation of glucose.

E) Maintains all hormone concentrations at resting levels.

Question 6 — Analyzing | Substrate Selection | Difficult

Consider the following statements:

I. The relative contribution of carbohydrates and lipids to energy production may vary according to exercise intensity.

II. Exercise duration may modify the relative contribution of energy substrates.

III. Utilization of a particular substrate does not necessarily mean that other energy sources have completely ceased to contribute.

IV. Predominance of one metabolic pathway implies exclusive activity of that pathway.

Based on the principles of exercise metabolism, select the correct alternative.

A) I and II only.

B) I, II, and III only.

C) II and IV only.

D) III and IV only.

E) I, II, III, and IV.

Question 7 — Analyzing | Fick Principle and Cardiovascular Integration | Difficult

During progressive exercise, oxygen consumption increases to meet the increasing metabolic demand. Consider the conceptual relationship:

VO₂ = cardiac output × arteriovenous O₂ difference

Based on this relationship, consider the following statements:

I. The increase in VO₂ during exercise may involve an increase in the amount of blood pumped by the heart per unit of time.

II. Changes in peripheral O₂ extraction may also contribute to the increase in VO₂.

III. Oxygen transport and utilization during exercise depend on the integration of central and peripheral mechanisms.

Select the correct alternative.

A) I only.

B) II only.

C) I and III only.

D) I, II, and III.

E) III only.

Question 8 — Analyzing | Ventilation and Acid–Base Balance | Difficult

As the intensity of an incremental exercise test increases, pulmonary ventilation also rises. At higher intensities, this ventilatory increase may become proportionally greater.

Considering the integration between metabolism and respiratory control, evaluate the following statements:

I. Ventilation contributes to maintaining the O₂ and CO₂ exchange required during exercise.

II. At high exercise intensities, changes related to acid–base balance may contribute to the increased ventilatory response.

III. Pulmonary ventilation is independent of the metabolic changes associated with increasing exercise intensity.

Which statements are correct?

A) I only.

B) II only.

C) I and II only.

D) II and III only.

E) I, II, and III.

Question 9 — Evaluating | Training Adaptation | Very Difficult

Following a period of aerobic training, a student showed improvements in VO₂max and physical performance. Three explanations were proposed:

I. The improvement may involve cardiovascular adaptations that enhance oxygen transport.

II. Peripheral skeletal muscle adaptations may also contribute to more efficient oxygen utilization.

III. The improvement must necessarily be attributed to a single isolated physiological adaptation.

Considering the integrated nature of physiological adaptations to training, which judgment is most appropriate?

A) Only statement III is physiologically consistent.

B) Statements I and II are consistent, whereas statement III represents an overly reductionist interpretation of the adaptive process.

C) Statement I is correct, whereas statements II and III are incorrect.

D) Statement II is correct, whereas statements I and III are incorrect.

E) All statements are correct because they represent independent mechanisms.

Question 10 — Evaluating | Systems Integration | Very Difficult

During incremental exercise, several responses occur simultaneously, including increased skeletal muscle ATP demand, changes in substrate utilization, hormonal responses, increased cardiac output, and ventilatory adjustments.

A student provides the following interpretation:

“These responses represent independent phenomena that occur simultaneously only because exercise affects several organs.”

Considering the principles of Exercise Physiology, evaluate this interpretation.

A) The interpretation is appropriate because physiological systems operate independently during exercise.

B) The interpretation is partially appropriate because functional integration occurs only between the cardiovascular and respiratory systems.

C) The interpretation is inappropriate because metabolic, hormonal, cardiovascular, and respiratory responses are functionally integrated to meet the demands of exercise and contribute to the maintenance of homeostasis.

D) The interpretation is appropriate because homeostatic regulation ceases when energy demand increases.

E) The interpretation is inappropriate only because energy metabolism does not change during exercise.
